# Cardiovascular protection by DPP-4 inhibitors in preclinical studies: an updated review of molecular mechanisms

**DOI:** 10.1007/s00210-022-02279-3

**Published:** 2022-08-10

**Authors:** Esraa M. Zakaria, Walaa M. Tawfeek, Mohamed H. Hassanin, Mohammed Y. Hassaballah

**Affiliations:** 1 Department of Pharmacology, Faculty of Pharmacy, Zagazig University, Zagazig, 44519 Egypt; 2 Faculty of Pharmacy, Zagazig University, Zagazig, 44519 Egypt

**Keywords:** Dipeptidyl peptidase 4 (DPP4), DPP4 inhibitors, Gliptins, Glucagon-like peptide-1 (GLP-1), Diabetes, Cardiovascular disease

## Abstract

Dipeptidyl peptidase 4 (DPP4) inhibitors are a class of antidiabetic medications that cause glucose-dependent increase in incretins in diabetic patients. One of the two incretins, glucagon-like peptide-1 (GLP-1), beside its insulinotropic activity, has been studied for extra pancreatic effects. Most of DPP4 inhibitors (DPP4i) have been investigated in in vivo and in vitro models of diabetic and nondiabetic cardiovascular diseases including heart failure, hypertension, myocardial ischemia or infarction, atherosclerosis, and stroke. Results of preclinical studies proved prominent therapeutic potential of DPP4i in cardiovascular diseases, regardless the presence of diabetes. This review aims to present an updated summary of the cardiovascular protective and therapeutic effects of DPP4 inhibitors through the past 5 years focusing on the molecular mechanisms beneath these effects. Additionally, based on the results summary presented here, future studies may be conducted to elucidate or illustrate some of these findings which can add clinical benefits towards management of diabetic cardiovascular complications.

## Introduction

Type 2 diabetes mellitus (DM) is associated with various cardiovascular (CV) complications including hypertension, ischemic heart disease, heart failure, and atherosclerosis. Diabetic patients tend to develop cardiovascular disease at a younger age and at higher incidence than non-diabetic patients (Chen et al. 2016; Leon and Maddox 2015). In addition to the deleterious effects of hyperglycemia, studies proved that diabetic patients exhibit an impaired response to intestinal hormones called incretins compared to individuals with normal blood glucose level. This sheds lights on a new family of antidiabetic medications, the dipeptidyl peptidase 4 inhibitors (DPP4i) or gliptins, which target incretin hormones. Several DPP4i are commercially available including alogliptin, linagliptin, saxagliptin, sitagliptin, anagliptin, vildagliptin, teneligliptin, and omarigliptin, in addition to several others which are under development.

Incretins are hormones released by the intestinal mucosa to stimulate insulin secretion following oral nutrient intake. Humans express two types of incretins: glucagon-like peptide-1 (GLP-1) and glucose-dependent insulinotropic peptide (GIP). GLP-1 is secreted by the neuroendocrine L cells in the ileum, while GIP is secreted by K cells in the duodenum and jejunum. Both hormones promote insulin release from pancreatic β-cells by activation of intracellular cAMP. They also stimulate β-cell proliferation and inhibit apoptosis, which increases β-cell mass (Drucker 2006). In addition, GLP-1, but not GIP, inhibits gastric emptying, food intake, and pancreatic release of glucagon, the hormone which stimulates hepatic glucose production.

GLP-1 has extra pancreatic effects and its receptor is expressed on many sites including the brain, intestine, lung, adipose tissue, myocardium, and vascular smooth muscle cells (Bullock et al. 1996). On the other hand, GIP has no major role in the cardiovascular system (Nauck and Meier 2018). GLP-1(7–36) is the active form that is degraded to an inactive form, GLP-1 (9–36), within 1 to 2 min by the enzyme DPP4 (Mafong and Henry 2009). DPP-4 is a widely expressed enzyme that exists in 2 forms, an extracellular protein that, when activated, initiates intracellular signal transduction pathways independent of its enzymatic activity, as well as a circulating soluble form which is enzymatically active (Drucker 2006).

Current developments in incretin research have demonstrated a significant role of incretins in cardiovascular pathology. This comprises 2 approaches. Firstly, the role of GLP-1 which is mediated through receptor-dependent and receptor-independent mechanisms. GLP-1 signaling seems to play a role in cardiac development, because mice with a targeted gene deletion of the GLP-1 receptors showed enlarged hearts (Gros et al. 2003). Diebold et al. (2018) demonstrated a significant role of GLP-1 secretion for left ventricular contractility during myocardial infarction. Mechanistically, it was shown that DPP-4 inhibition increased AMP-activated protein kinase (AMPK) activity and stimulated the mitochondrial respiratory capacity of non-infarcted myocardial tissues.

Secondly, it is now known that DPP-4 also cleaves several other peptides, some of which have direct actions on the cardiac and vascular cells. Hence, DPP4 inhibition provides favorable cardiovascular outcomes independent of GLP-1. DPP-4 is responsible for degradation of B-type natriuretic peptide (BNP, Brandt et al. 2006), stromal cell-derived factor 1α (SDF-1α, Zaruba et al. 2009), and substance P (Wang et al. 1991). These findings suggest DPP4i as potential cardioprotective agents in diabetic and non-diabetic patients specially when considering that DPP4i have no risk of hyperglycemia or weight gain.

This review presents an updated overview on the cardiovascular protective effects of DPP4i in experimental preclinical studies during the past 5 years. In addition, this review describes and evaluates the underlying molecular mechanisms for these effects.

## Methodology

### Literature search

To gather the most recent experimental studies on DPP4i in animal models of cardiovascular disease, a literature search was performed in “PubMed” and “Google Scholar” search engines up to 20 February 2022. The keywords “DPP4 inhibitors or gliptins or linagliptin or vildagliptin or saxagliptin or sitagliptin or alogliptin or anagliptin or teneligliptin or omarigliptin,” were matched with “cardiovascular or myocardial ischemia reperfusion or myocardial infarction or heart failure or atherosclerosis or stroke,” and with “experimental animals.” We included studies published in trustful scientific journals starting from 2017 and thereafter. To ensure a comprehensive search, we also included some related articles from references. Articles were carefully read, and data related to disease models, DPP4i dose, treatment duration, and main findings as well as the underlying molecular mechanisms were summarized in a tabular form.

### Figure creation

Figure 2 was created using BioRender software tool.

## Results

Results of our search of DPP4i cardiovascular effects are summarized in a tabular form. Results are classified based on the CV disease then the type of gliptin (Table 1).

**Table 1:** Protective effects of DPP4i in various cardiovascular disease models in experimental animals

No	Gliptin, dose, route, and treatment duration		Model and subject	Main finding after gliptin treatment (and molecular mechanism)	Author
*Myocardial ischemia reperfusion (I/R)*					
1	Sitagliptin	300 mg/kg, PO 3 days prior to I/R	Myocardial I/R by coronary ligation in male Wistar rats	↓Elevated cardiac enzymes, infarct size, and apoptosis markers ↑Natriuretic peptide and cGMP Improved hemodynamic parameters (HR and LVDP)	(Abbas et al. 2018)
2		50 mg/kg/day, PO for 2 wks	Ex vivo I/R with various durations	↓Infarct size and DPP4 activity ↑GLP-1, e-NOS expression, TRPV-1 level, and TRPC-1 expression	(Al-Awar et al. 2018)
3		Sitagliptin or Saxagliptin 0.6, 0.45 mg/kg/day, respectively, IP for 3 wks	Evaluation of diabetic rat hearts with or without global ischemia (30 min)	***Both gliptins:*** improved in vivo hemodynamic parameters ↓Cardiac apoptosis (Bcl-2 and TUNEL staining) and necrosis ↓Cardiac troponin T Improved coronary circulation (saxa < < sita)	(Bradic et al. 2021)
4		10 mg/kg/day, PO for 4 wks started with isoproterenol dosing	Myocardial ischemia by isoproterenol injection for 2 days in diabetic rats	Improved cardiac conductivity and structural changes ↑VEGF, CD34, IGF-1 ↓Cardiac enzymes, inflammation and COX activity	(Khodeer et al. 2019)
5		10 mg/kg/day, PO, post induction for 21 days	Myocardial infarction in diabetic C57BL/6 mice	↓Infarct size, fibrosis Improved the impaired autophagy (↑ LC3II and P65 levels)	(Gu et al. 2018)
6	Vildagliptin	80 mg/kg/twice daily for 2 days Alone or combined with Granulocyte colony stimulating factor (G-CSF)	I/R in mice by ligation of the left anterior descending (LAD) coronary artery	↓Infarct size ↑Myocardial homing of circulating CXCR4^+^ stem cells and angiogenesis ↑SDF-1α mRNA expression	(Li et al. 2019)
7		6 mg/kg/day, PO for 1 month before I/R combined with ischemic preconditioning	In vitro regional I/R on Langendorff apparatus Regional myocardial ischemia of diabetic hearts by ligation of left coronary artery	Improved diabetes-mediated inhibition of left ventricular pressure and contractility The combination ↓Infarct size The combination ↓Infarct size, ↓overactivated autophagy markers (LC3B-II and LC3BII/LC3BI and p62), ↓Mitochondrial ROS	(Bayrami et al. 2018b) (Bayrami et al. 2018a)
8		6 mg/kg/day PO for 5 wks prior to I/R Alone or combined with ischemic preconditioning	Ex vivo regional I/R of isolated diabetic rat hearts by Langendorff apparatus	↓Gene expression of autophagy marker LC3-II ↑Gene expression of mitochondrial fusion marker mfn2	(Pirzeh et al. 2019)
9		3 mg/kg/day PO, for 4 wks	I/R in ovariectomized rats I/R by ligation of LAD of prediabetic rat hearts	↑% of LVEF ↓Infarct size, arrhythmia score, oxidative stress, and apoptosis	(Sivasinprasasn et al. 2017) (Tanajak et al. 2018)
10	Anagliptin	300 mg/kg/day in drinking water 5 days before MI	Myocardial ischemia by ligation of LAD in diabetic rat hearts	↓Infarct size ↑HMGB1 plasma levels, angiogenesis Normalized VEGF expression	(Sato et al. 2017)
11	Linagliptin	10 mg/kg/day in drinking water for 8 wks	Obesity-induced myocardial ischemia in mice	↑Angiogenesis (EGR-1) Cardiac citrulline and creatine levels	(Suda et al. 2017)
12		5 mg/kg/day in drinking water for 4 wks after induction of MI	MI by permanent LAD ligation in congenital DPP-4-deficient Fischer 344 rats	Improved LV diastolic function, ↓fibrosis (gene expression of collagen, TGF-β1), and inflammation (gene expression of MCP-1 and MMP-2)	(Yamaguchi et al. 2019)
13		3 mg/kg/day combined to empagliflozin for 7 days prior to I/R and continued for 28 days post I/R	Myocardial ischemia by 30 min ligation of LAD in diabetic mice	↓Fibrosis and preserved systolic function	(Ideishi et al. 2021)
14		83 mg/kg of chow for 1 wk before I/R and continued after surgery in the myocardial infarction model	Model 1: I/R ischemia (30 min) then reperfusion (24 h) in diabetic mice Model 2: MI by permanent coronary artery occlusion	↓ASC, NALP3, IL-1β, IL-6, Collagen-1, and Collagen-3, TNFα ↓TLR4 expression with downstream upregulation of Let-7i and miR-146b levels ↓Nlrp3/ASC infammasome by p38 activation with downstream upregulation of miR-146b levels	(Birnbaum et al. 2019)
15	Linagliptin	9 nmol/L linagliptin, 817 nmol/L sitagliptin, 11 ng/mL alogliptin, 24 ng/mL saxagliptin, or 47 ng/mL 5-hydroxy saxagliptin	In vitro myocardial I/R of C57BL/6 mice in perfused heart technique	Linagliptin ↑(LVDP), dP/dtmax, ↓dP/dtmin, and phospho-protein phospholamban (Ser16) levels. Indirectly activated intracellular signaling in cardiomyocytes by ↑serine473 phosphorylation of Akt and serine1177 phosphorylation of eNOS	(Batchu et al. 2020)
Cardiomyopathy					
16	Sitagliptin	100 mg/kg/day, PO for 2 wks	Diabetes-associated cardiac injury	↓Bcl-2-associated X protein, caspase-3, apoptosis-inducing factor expression ↑Bcl-2, HSP-70 in left ventricular tissue	(Mansour et al. 2021)
17		15 mg/kg/day for 12 wks	Obesity-induced cardiac dysfunction in female mice	Alleviated diastolic dysfunction, ↓mTOR/S6K1 activation	(Qiao et al. 2018)
18		10 mg/kg/day, PO, for 12 wks	Cardiomyopathy in Zucker diabetic fatty rats	Improved dyslipidemia, ejection fraction, and fractional shortening ↓Nitrosative stress and reversed the inhibited autophagy	(Zhou et al. 2018)
19		10 mg/kg/day, PO for 21 days (either alone or combined with quercetin)	Doxorubicin-induced cardiotoxicity in male adult Wistar rats	↓Level of troponin, LDH, CK, CRP, cholesterol, LDL, TG, plasma atherogenic index ↑Total antioxidant capacity	(Aziz 2021)
20		200 mg/kg/twice a day, for 8 wks	Nephrectomy-induced cardiac remodeling In male Wistar rats (5/6 nephrectomy)	↓Fibrosis and hypertrophy ↓Isovolumic relaxation time ↓Cardiac content of Ang II but ↑Ang-(1–7) ↓Cardiac Ang II but ↑Ang-1–7	(Beraldo et al. 2019)
21		10 mg/kg/day, PO, for 8 wks	Hypertension in Dahl salt-sensitive rats (induced by a high-salt diet for 5 weeks)	Improved diastolic function ↓Plasma BNP ↓TNF-α, IL-6, CCL2, and NF-κB ↓NOX2, levels of DHE oxidation ↓Collagen deposition and TGF-β level	(Esposito et al. 2017)
22		22.6 mmol/kg or the new DPP4i, LASSBio-2124 (22.6 mmol/kg), by oral gavage once a day for 2 wks	Diabetes-induced cardiac dysfunction in male Wistar rats	↓Cholesterol, TG levels, systolic, and diastolic left ventricular dysfunction LASSBio-2124 reversed the impairment of vascular reactivity	(Alves et al. 2019)
23	Linagliptin	83 mg/kg for 16 wks added to western diet	Obesity-induced cardiac dysfunction in female C56Bl/6 J mice (by high fat and simple sugar-rich diet)	↓NF-κB, AP-1, and p-38 MAPK activation ↓Cardiac nitrative and oxidative stress by ↓ MDA/4HNE levels ↓TRAF3IP2 protein and gene expression ↓Cardiac fibroblast (CF) activation and migration, collagens I and III expression ↓Hypertrophy marker 70 S6 kinase1 ↓Diastolic and systolic dysfunction	(Aroor et al. 2017)
24		10 mg/kg, IV, at 1 h after surgery	Obesity and insulin resistance–induced cardiac dysfunction in male C57BL/6 mice	↓Cardiac dysfunction associated with CLP-sepsis in diabetic mice ↓IL-6, KC, IL-10, and TNF-α ↓MPO and NAG activities in the lungs ↓Serum creatinine, urea, and ALT levels	(Al Zoubi et al. 2018)
25		83 mg/kg in chow diet for 4 weeks	Cardiac dysfunction in obese ZSF1 rats (homozygous for the leptin receptor mutation)	↓Left ventricular stiffness and improved relaxation (↓mitral valve deceleration time) ↓Transcript levels of Col1a1, Col3a1, and Timp1 leading to reduction of total, perivascular, and interstitial cardiac fibrosis	(Cuijpers et al. 2021)
26	Vildagliptin	3 mg/kg/day, PO, for 28 days	High fat diet–induced cardiac dysfunction in male Wistar rats	↑Bcl-2, ↓Bax, and cleaved caspase 3 expression	(Tanajak et al. 2017)
27		3 mg/kg/day alone or combined with low-dose testosterone, PO for 28 days	Castrated obese insulin resistant male rats	↑LVEF ↓LF/HF ratio, systolic, and diastolic BP ↓Cardiac mitochondrial ROS, mitochondrial membrane depolarization, and swelling ↑Expression of PGC-1α, CPT-1, and OPA-1 ↓p-Drp1ser616/Drp1 protein expression and TUNEL + cells	(Arinno et al. 2019)
28		15.17 mg/kg/day, PO, for 10 wks	Cardiac dysfunction in wild-type C57BL/6 J and miR-21 knockout mice by treatment with HFD/STZ	↑E/A value, LVEF, and fractional shortening, expression of Cx43 →improved cardiac function ↓Cardiac fibrosis in diabetic mice →maintained cell–cell communication and cardiac function	(Li et al. 2021)
29		10 mg/kg/day, PO for 9 wks	Diastolic dysfunction in Dahl salt–sensitive rats	↓LVEDP, LV distensibility index, LV interstitial fibrosis ↓Plasma renin activity and aldosterone concentrations	(Nakajima et al. 2019)
30		50 mg/kg/day for 4 wks	Myocardial pressure overload in male C57BL/6j mice (produced by constricting the transverse aorta)	↓Myocardial FGF21 expression via Sirt1 expression →↓Cardiac hypertrophy and dysfunction ↑FDG (glucose analog) uptake and BMIPP (fatty acid analog) uptake	(Furukawa et al. 2021)
31	Saxagliptin	10 mg/kg/ day, PO, for 8 wks	Diabetic cardiomyopathy in mice	↓Myocardial lipid accumulation, oxidative stress, apoptosis, and cardiac remodeling	(Wu et al. 2018)
32		10 mg/kg/day, PO in peanut butter for 2 wks, started after Ang II treatment	Ang II-induced cardiac dysfunction in male C57BL/6 J mice (Ang II dose = 500 ng/kg/min)	Improved diastolic function (normalization of early-to-late septal annulus motion in diastole and a tendency to decrease isovolumic relaxation time) Prevented Ang II–induced cardiac periarterial fibrosis by ↓collagen I mRNA expression and cardiac periostin expression ↓Cardiac CD11c messenger RNA and cardiac CD8 gene expression and memory CD45, CD8, CD44 lymphocytes, TLR4, NFkB, AP-1	(Brown et al. 2017)
33	Alogliptin	20 mg/kg, day, PO, for 8 wks	Cardiac dysfunction in SHR male rats	↓Systolic and diastolic BP ↓Cardiomyocyte size and collagen expression ↓Expressions of RhoA and ROCK2 and the phosphorylation of the ROCK2 substrates MLC and MYPT1 → reduction of myocardial hypertrophy via the cAMP/PKA/RhoA/ROCK2 signaling	(Fan et al. 2020a)
34	Teneligliptin	10 mg/kg/day started after induction of hypertension Early treatment for 12 wks (from wk 6 to wk 18) Late treatment for 8 wks (from wk 10 to wk 18)	Cardiomyopathy in Dahl salt–sensitive rats	Prevented cardiomyocyte fibrosis, concentric hypertrophy, and development of heart failure	(Yamamoto et al. 2018)
35		30 mg/kg/day, in drinking water for 1 wk	Ang II-Induced cardiac hypertrophy	↓Ang II-induced increases in Nox4-HDAC4 axis in cardiomyocytes via a GLP-1 receptor-dependent manner	(Okabe et al. 2020)
*Atherosclerosis and other vasculopathies*					
36	Alogliptin	20 mg/kg/day, PO for 8 wks	Vascular remodeling: In vivo: in SHR In vitro: rat aortic smooth muscle cells exposed to Ang II	↓Proliferation, ECM degradation, downregulation of MMP-1, ERK1/2, NF-κB	(Fan et al. 2020b)
37	Saxagliptin	10 mg/kg/day in drinking water for 12 wks	In vivo*:* aged rats In vitro*:* H2O2-induced senescent human umbilical vein endothelial cells	↑Expression and phosphorylation of AMPK-α, SIRT1, Nrf2	(Chen et al. 2020)
38		10 mg/kg/day, PO, started 1 wk after aortic banding and continued for 23 wks	Coronary conduit vascular stiffness induced by aortic banding in miniature swine	Normalized coronary vascular stiffness by ↓AGEs, NF-κB, and nitrotyrosine levels	(Fleenor et al. 2018)
39	Vildagliptin	50 mg/kg/day, PO, for 4 wks after induction of DM	DM-induced vascular endothelial dysfunction in wild or TRPV4 − / − diabetic mice	Improved endothelial dysfunction by direct activation of TRPV4 →↑extracellular calcium uptake in endothelial cells→↑ AMPK/SIRT1 pathway	(Gao et al. 2020)
40		35 mg/kg/day, PO, started the next day to ligation and continued for 4 wks	Artery stenosis by carotid artery ligation in a genetic mouse model of DM	↓Endoplasmic reticulum stress/NF-κB pathway	(Ji et al. 2019)
41		3 mg/kg/day, in drinking water, for 6 wks	Doxorubicin-induced vascular senescence	Improved vascular relaxation ↓Senescence markers, p16^Ink4a^, and p27^Kip1^ expression ↓IL-6 and IL-8	(Mišúth et al. 2021)
42		Low dose 10 mg/kg/day High dose 20 mg/kg/day, PO for 12 wks	Aortic endothelial dysfunction in diabetic rats	miRNA regulation to inhibit Ccl2 expression and to increase BDNF and Pdk1 expression in the aorta (↓inflammation and apoptosis)	(Zhang et al. 2021)
43	Sitagliptin	50 mg/kg, PO for 1 month after induction	Vascular inflammation induced in vivo by hypercholesterolemia in mice and in vitro by TNF-α-stimulated human umbilical vein endothelial cell line	In both models: ↑SIRT6 expression ↓The expression of MCP-1, IL-6, and IL-1β which is partially SIRT6-dependent and partially due to ↓ ROS	(He et al. 2019)
44		2.5, 10 mg/kg/day, PO, for 90 days	Allograft vasculopathy model using the PVG/Seac rat thoracic aorta graft to ACI/NKyo rat abdominal aorta	↓BNP and HMGB1 levels ↑GLP-1 activity and SDF-1α expression	(Lin et al. 2021)
45		20, 40, and 80 mg/kg/day, PO for 28 days	Pulmonary arterial remodeling in rats	Improved hypertrophy of pulmonary arterial medial layer, ↓intracellular inflammation, chronic hypoxia-induced pulmonary hypertension	(Xu et al. 2018)
46	Anagliptin	30 mg/kg/twice daily, PO for 12 wks	Atherosclerosis induced in ApoE − / − mice by HFD and stress	Adiponectin-dependent ↓ atherosclerotic lesion	(Lei et al. 2017)
47	Linagliptin	83 mg/kg of chow for 4 wks	Vascular remodeling in male diabetic rats and nondiabetic Goto-Kakizaki rats	Improve total relaxation by ↑NO and vasodilation Significantly improved cerebral perfusion in the diabetic rats Reversed vascular remodeling (↓ media thickness and media-to-lumen ratio)	(Hardigan et al. 2016)
Cerebral ischemia/stroke					
48	Vildagliptin	2.5, 5, 10 mg/kg for 3 wks prior to stroke	Cerebral ischemia in rats by left middle cerebral artery occlusion (MCAO)	Improved neurological deficit score, locomotor activity, and motor coordination, ↑Antioxidants and mTOR contents in brain	(El-Marasy et al. 2018)
49			In vitro hypoxia/reoxygenation model in isolated rat primary cardiac microvascular endothelial cells	↓Activation of the p38/NF-κB signaling in hypoxia/reoxygenation-induced cardiac microvascular endothelial cells	(Fan et al. 2020c)
50	Linagliptin	10 mg/kg/day, PO, for 8 wks started 3 days after stroke	Stroke in T2D/obese mice (induced by transient MCAO)	↓Post-stroke neuroinflammation, normalized microglia/macrophages activation Improved neuroplasticity (by preserving soma volume of PV + interneurons and ↑stroke-induced neuroblast formation)	(Augestad et al. 2020)
51		10 mg/kg/day for 1 wk beginning the day of stroke onset, then 83 mg/kg in chew diet for 2 more wks	Stroke in mice (induced by transient MCAO)	Improved functional stroke outcome by boosting SDF-1α/CXCR4 pathway	(Chiazza et al. 2018)
52		10 mg/kg, PO	Focal cerebral ischemic stroke in adult male mice	Activated Akt/mTOR signaling pathways ↑The anti-apoptotic protein Bcl-2 ↓The pro-apoptotic protein Bax	(Zhang et al. 2020)
53	Teneligliptin	60 mg/kg/day, for 20 wks	Atherosclerosis using apolipoprotein-E-deficient (ApoE^−/−^) mice	↓Inflammation (↓ expression of TNF-α and MCP-1) in abdominal aorta ↓Expression of adipocyte Nox-4 Improved endothelium-dependent vasodilation and ↓oxidative stress	(Salim et al. 2017)

***4HNE***, 4-Hydroxynonenal; ***ACE2***, Angiotensin-converting enzyme 2; ***AGEs***, Advanced glycation end products; ***ALT***, alanine transaminase; ***AMPK-α***, AMP-activated protein kinase-α; ***Ang II***, Angiotensin II; ***ANP***, atrial natriuretic peptide; ***BNP***, B-type natriuretic peptide; ***AP-1***, activating protein-1; ***ASC***, 1-Aminocyclopropane-1-carboxylic acid synthase; ***AT1R***, Angiotensin II type 1 receptor; ***Bcl-2***, B-cell lymphoma 2; ***BDNF***, brain-derived neurotrophic factor; ***cAMP***, cyclic adenosine monophosphate; ***Ccl2***, C–C motif chemokine ligand 2 (aka MCP-1 monocyte chemoattractant protein); ***CK***, creatine phosphokinase; ***Col1a1***, collagen, type I, alpha 1; ***COX***, Cyclooxygenase; ***CPT-1***, Carnitine palmitoyl transferase 1; ***CRP***, C-reactive protein; **Cx43**, connexin 43; **CXCR4**, C-X-C motif chemokine receptor 4; ***DHE***, Dihydroethdium; ***E/A ratio***, the ratio of early (E) to late (A) ventricular filling velocity; ***ECM***, extracellular matrix; ***EGR-1***, early growth response protein 1; ***e-NOS***, Endothelial nitric oxide synthase; ***ERK1/2***, extracellular regulated protein kinase ½; ***FDG***, Fluorodeoxyglucose; ***FGF21***, fibroblast growth factor 21; ***HDAC4***, Histone deacetylase 4; ***HMGB1***, high mobility group box 1; ***HR***, heart rate; ***HSP-70***, heat shock protein 70; ***I/R***, ischemia/reperfusion; ***IGF-1***, insulin-like growth factor 1; ***IL-1β***, Interleukin-1β; ***KC***, Keratinocyte chemoattractant; ***LC3I***, Microtubule-associated protein light chain I; ***LDH***, lactate dehydrogenase; ***LDL***, low-density lipoprotein; ***LVDP***, left ventricular developed pressure; ***LVEDP***, left ventricular end diastolic pressure; ***LVEF***, left ventricular ejection fraction; ***MAPK***, mitogen-activated protein kinase; ***MDA***, Malondialdehyde; ***MI***, myocardial infarction; ***MMP-2***, matrix metalloproteinase-2; ***MPO***, Myeloperoxidase; ***mTOR***, mammalian target of rapamycin; ***NAG***, N-acetyl-β-D-glucosaminidase; ***Nlrp3***, (aka NALP3) NLR family pyrin domain containing 3; ***NF-κB***, nuclear factor kappa B; ***Nrf2***, nuclear factor erythroid 2-related factor 2; ***Pdk1***, pyruvate dehydrogenase kinase 1; ***PGC-1α***, Peroxisome proliferator-activated receptor-gamma coactivator 1α; ***PKA***, protein kinase A; ***ROCK***, Rho-associated protein kinase 2; ***ROS***, reactive oxygen species; ***S6K1***, S6 kinase-1; ***SDF-1α***, stromal cell–derived factor-1α; ***SHR***, spontaneously hypertensive rats; ***SIRT1***, sirtuin-1; ***TG***, Triglycerides; ***TGF-β1***, transforming growth factor beta 1; ***Timp1***, tissue inhibitor of metallopeptidase-1; ***TLR4***, Toll-like receptor-4; ***TNF-α***, tumor necrosis factor-α; ***TRAF3IP2***, TRAF3 (TNF receptor-associated factor) interacting protein 2; ***TRPC-1***, transient receptor potential cation-1; ***TRPV-1***, transient receptor potential channel vanilloid-1; ***TUNEL***, terminal deoxynucleotidyl transferase dUTP nick end labeling; ***VEGF***, vascular endothelial growth factor; ***β-MHC***, beta-myosin heavy chain

## Discussion

This review covers the recent research of the cardiovascular (CV) protective potential of DPP4 inhibitors (DPP4i) during the past 5 years. It is believed that the protective effects of DPP4i are maintained through 2 approaches, (I) Glucagon-like peptide-1 (GLP-1)–mediated mechanisms and (II) conservation of some peptides that are physiologically degraded by the DPP4 enzyme. Researchers reported that DPP4 is responsible for degradation of B-type natriuretic peptide (BNP), stromal cell–derived factor 1α (SDF-1α), and substance P. Gliptins effectively mitigate the deleterious effects of postprandial hyperglycemia on oxidative stress, inflammation, and CV remodeling. However, in models of CV disease in non-diabetic animals, gliptins show protective effects through various pathways that will be discussed here.

**Fig. 1:**
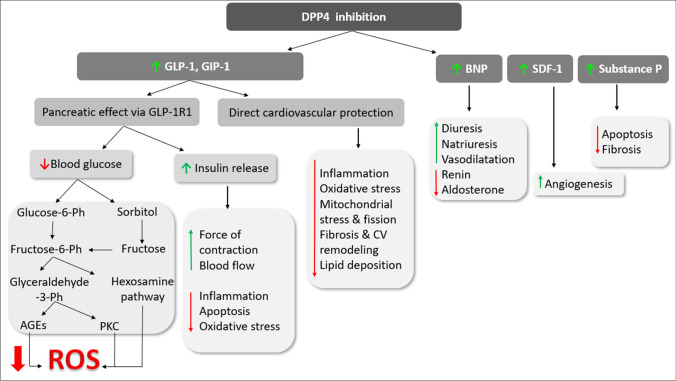
Biological consequences of DPP4 inhibition

GLP-1 deficiency is a consequence rather than a cause of diabetes as GLP-1 secretion is decreased in hyperglycemia. Also, DPP4 activity is increased in diabetes, both type 1 and 2, and is negatively correlated with adiponectin levels (Vollmer et al. 2009). The effect of DPP4 antagonism was studied in models of myocardial ischemia/reperfusion (I/R) and infarction in normoglycemic and hyperglycemic animal models of CV disease by genetic deletion of DPP4 or by using DPP4i. Both approaches resulted in improved cardiac function and hemodynamics (Sauvé et al. 2010). These promising finding may be attributed to (I) increased GLP-1 level which exerts its effects through receptor-dependent as well as receptor-independent mechanisms. (II) DPP4 inhibition preserves some peptides that have advantages on CV function in various disease conditions (see Fig. 1).

### I-DPP4 inhibition increases GLP-1

It is well known that hyperglycemia underlines diabetic cardiovascular complications through various pathways. Glucotoxicity increases reactive oxygen species (ROS) production through many mechanisms including polyol and hexosamine pathways, increased advanced glycation end products (AGEs) production, activation of protein kinase C (PKC) and poly(ADP-ribose) polymerase-1 (PARP-1) enzyme (Mapanga and Essop 2016). Increased ROS production triggers a closed cycle of myocardial and vascular inflammation and oxidative stress leading to apoptosis, endothelial dysfunction, and cardiomyopathies. In healthy individuals, GLP-1 is secreted by the neuroendocrine L cells in the ileum in response to food ingestion and stimulates pancreatic beta cells to release insulin. Postprandial insulin augmentation helps regulate blood glucose with minimal hypoglycemic effect. As GLP-1 is impaired in diabetes, inhibiting its degradation helps decreasing blood glucose in diabetic patients and consequently accentuate the rush through these oxidative and nonoxidative glucotoxicity pathways. In addition, enhanced insulin secretion improves cardiac function and increase inotropy, regulate blood flow to myocardial tissue, and exerts anti-inflammatory, antioxidant, and antiapoptotic effects (Ng et al. 2012).

Beside the glycemic effect of GLP-1, it exerts direct cardiovascular protection. Since the discovery of GLP-1 receptor expression in the cardiac muscle, cardioprotective role of GLP-1 was extensively studied and was proved. Infusions of GLP-1 in animal models and human subjects with heart failure have demonstrated significant improvement in cardiac parameters (Ban et al., 2008). In patients with type 2 diabetes (T2DM), GLP-1 infusion significantly improved endothelial function, irrespective of changes in insulin sensitivity (Bullock et al. 1996). Moreover, infusion of GLP-1 in patients with T2DM and established coronary artery disease significantly improved endothelial dysfunction as measured by flow-mediated vasodilation (Nyström et al. 2004). Observations suggest that part of the cardioprotective and vasodilatory effects of GLP-1 on myocardial metabolism is direct, insulin-independent, and GLP-1-receptor–independent (Ban et al. 2008). A study by Gros et al. (2003) on GLP-1 receptor knock out mice (GLP-1R^−^/^−^) showed that lack of GLP-1R is associated with decreased resting heart rate and increased left ventricular end diastolic (LVED) pressure and LV thickness compared with CD-1 wild-type controls. In addition, GLP-1R deletion resulted in impaired LV contractility and diastolic function after insulin or epinephrine administration. In another way, a study on a mouse model of I/R found that GLP-1(9–36), the primary metabolite of GLP-1, has nearly identical results, suggesting the presence of an alternative signaling mechanism for GLP-1 and its metabolite independent of its known receptor.

### II-DPP4 inhibition preserves physiologically active cardioprotective peptides

Beside GLP-1, other peptides are substrates of DPP4 enzyme including atrial natriuretic peptide (ANP), brain natriuretic peptide (BNP), stromal cell–derived factor-1α (SDF-1α), and substance P. ANP is synthesized in the atria while BNP is produced by heart ventricles. They act locally and systemically to exert several biological functions including diuresis, natriuresis, and vasodilatation as well as inhibition of renin and aldosterone secretion (Nishikimi et al. 2006). Physiological levels of ANP and BNP are low but they increase as a compensatory mechanism in heart failure. The active form, BNP(1–32), is degraded by DPP4 by removing the two N-terminal amino acids (serine and proline) to produce BNP(3–32), which has reduced biological activity. Elevated levels of NPs were proved in hyperglycemia and decrease by improved glycemic control (Dal et al. 2014). However, in cardiac pathology, it seems that DPP4 is implicated in high levels of NPs as it was found that genetic deletion of DPP4 improved the elevated levels of ANP and BNP in rats subjected to myocardial ischemia/reperfusion (Ku et al. 2011). However, a recent meta-analysis of clinical studies on diabetic patients treated with DPP4i reported no significant effect of DPP4 on NP levels (Mu et al. 2022) and this finding needs further explanation.

SDF-1α is a chemokine that promotes cardiac homing of endothelial progenitor cells, to stimulate angiogenesis, which consequently improves myocardial perfusion. SDF-1α is a substrate of DPP4 enzyme and DPP4 inhibition preserves SDF-1α actions and promotes cardiac recovery after I/R (Pala and Rotella 2013), acute myocardial infarction (Li et al. 2019), or stroke (Chiazza et al. 2018). Another DPP4 substrate is substance P which has role in regulating heart rate and blood pressure. Substance P showed protective effects in some animal models of heart disease through inhibiting apoptosis, myocardial cell injury (Chen et al. 2022), and fibrosis (Widiapradja et al. 2021). On the contrary, in vitro studies reported that DPP4 inactivates fibrin by cleavage of fibrin a-chain leading to inhibition of fibrin polymerization and clot formation (Mentlein and Heymann 1982). This points to possible thrombolytic effect of DPP4 enzyme.

### Molecular pathways behind DPP4i effects on CV diseases

Diabetes is linked to a variety of cardiovascular diseases that lower the life quality of diabetic patients. Diabetic patients have many fold increase in the risk of atherosclerosis, myocardial ischemia, myocardial infarction, and heart failure. A common theme shared among these pathologies is massive ROS production that affects glucose metabolism and increase fatty acid oxidation. Additionally, ROS activates proinflammatory mediators, NRLP3 inflammasomes, and proatherogenic transcription factors. They also reduce mediators of tissue repair such as Nrf-2, sirtuin, and AMPK. Moreover, ROS stimulates mitochondrial fission leading to reduced efficiency of the mitochondrial electron transport chain and ATP synthesis, hence, myocardial ischemia and endothelial dysfunction. DPP4 mediates ROS production through several mechanisms of which glucotoxicity is major (see Fig. 1).

### *DPP4i inhibit oxidative stress *via* controlling glucotoxicity and lipotoxicity*

Studies showed that many gliptins significantly decreased ROS, RNS, DNA fragmentation, AGEs, and Nox4 and increased antioxidants in most of animal models of CV disease. In addition, a recent study by Wang et al. (2021) on liver inflammation in diabetic mice found direct ROS scavenging activity of sitagliptin (Wang et al. 2021). DPP4i work through several mechanisms that can improve myocardial perfusion. DPP4i preserve endothelial function by increasing eNOS phosphorylation and decreasing Ang II-mediated Nox-4 production. They decrease ischemia-induced damage by minimizing oxidative stress. They also increase the level of intracellular cAMP and activate cAMP-dependent protein kinase (PKA) and SDF-1α. Besides, they enhance eNOS activity with subsequent augmentation of endothelial-dependent vasodilatation and myocardial perfusion. Incretins might target postprandial lipid metabolism and thereby favorably influence several endothelial and cardiovascular functions. DPP4 release strongly correlates with adipocyte size and is considered risk factor for obesity (Pala and Rotella 2013). Several studies covered by this review found decreased serum TG and total cholesterol and LDL in models of obesity or insulin resistance. DPP4i improve insulin sensitivity which is mediated partially by Sirt-1 and Sirt-6 beside other mechanisms that collectively decrease oxLDL and saturated fatty acids.

### DPP4i improve CV inflammation

Preclinical studies show that DPP4i reduce myocardial inflammation via inhibition of cytokine release, monocyte activation, and chemotaxis. It is known that DPP4 significantly activate MAPK and NF-κB signaling pathway leading to vascular aging and dysfunction. Recent studies summarized in the “Results” section show that DPP4i control the release of many proinflammatory mediators such as NFkB, TNF-α, ILs, COX, MAPK, TLR4, CCl2, MCP-1, and MMPs. Inhibition of MMP activity maintains cellular architecture and prevents remodeling and fibrosis. DPP4 at least indirectly is implicated in endothelial and vascular smooth muscle cells structural remodeling and aging through inflammation and oxidative stress. In addition, ROS increases mitochondrial stress leading to energy shortage and subsequent CV senescence. The recent studies on animal models of myocardial infarction, I/R, and diabetes or obesity-induced cardiac remodeling reported that DPP4i significantly reduced hypertrophy, left ventricular interstitial, and periarterial fibrosis as evidenced by decreased expression of TGF-β, collagen, and components of cAMP/PKA/RhoA/ROCK2 pathway. Decreased fibrotic lesions in myocardial tissue will improve heart conductance and contractility.

### DPP4i inhibit cell death

DPP4i decrease overall cell death as evidenced by decreasing infarct size, serum cardiac enzymes (CK, LDH), and troponin T. In addition, they decrease proapoptotic markers MMP-2, HSP-70, and caspase-3 but increased antiapoptotic marker Bcl-2. In another way, DPP4i promote tissue repair mechanisms such as Sirt1,6 (vildagliptin, saxagliptin and sitagliptin) and Nrf-2 (saxagliptin). Also, DPP4 inhibition by sitagliptin or vildagliptin modulated the disturbed autophagy responses in CV disease (LC3II and P65 levels) and vildagliptin enhanced mitochondrial fusion (increased mfn-2 level). These findings are partly resulted from decreased oxidative stress and inflammation due to improved hyperglycemia but may also be attributed to direct action of GLP-1 on its receptor in the CVS.

### DPP4i improve CV hemodynamics

**Fig. 2:**
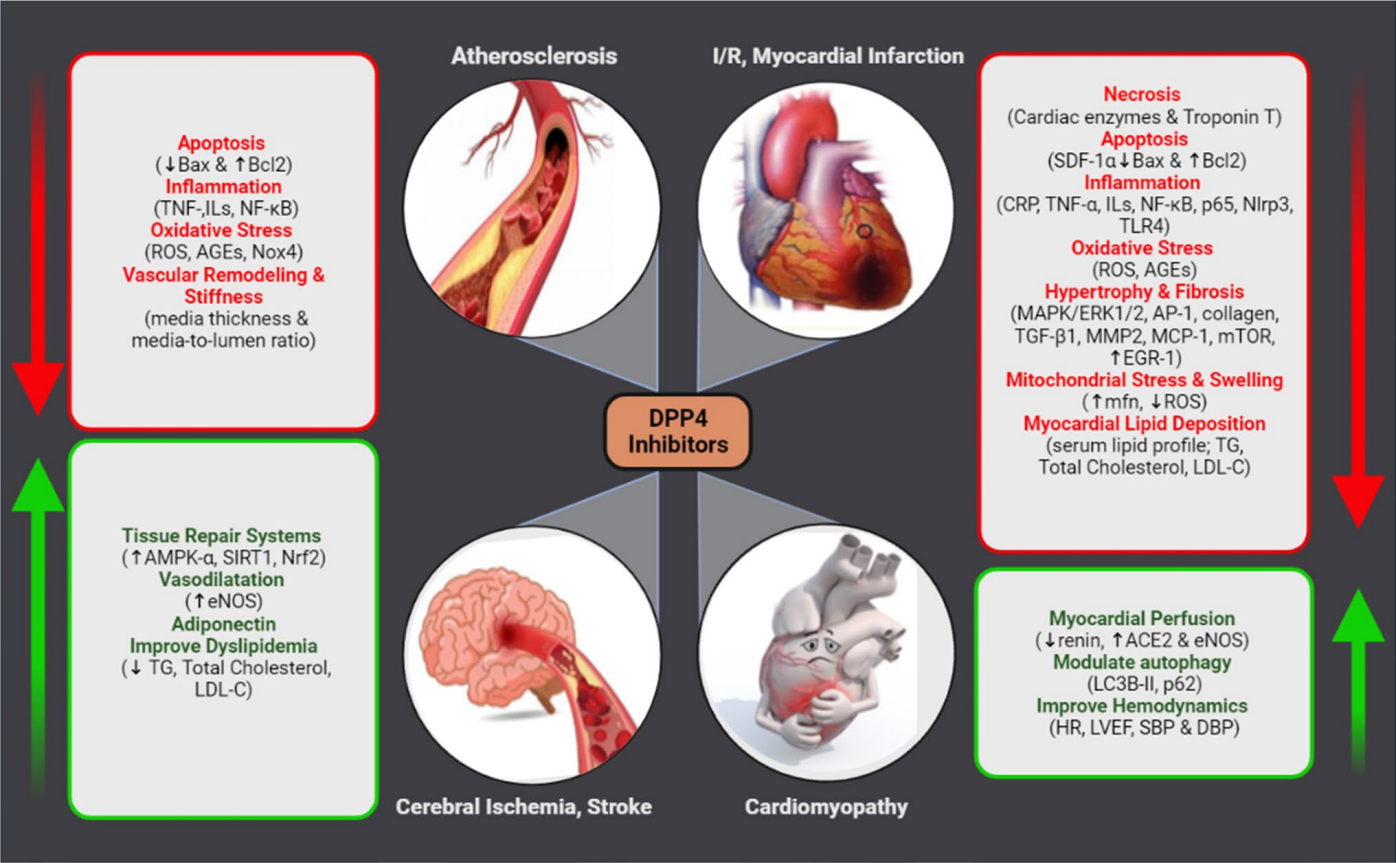
A summary of molecular mechanisms that underly cardiovascular protective effects of gliptins

Diabetic cardiomyopathies affect almost all parameters of CV hemodynamics with negative impact on both conductance and contractility. Diabetes in animal and human increases peripheral resistance due to endothelial dysfunction and atherosclerosis. Long-term increase in blood pressure attenuates cardiac output specially in the presence of other dependent or independent by CV pathologies. Results of our search in models of diabetes-, obesity-, or drug-induced cardiomyopathy showed that treatment with gliptins resulted in increased cardiac output and left ventricular ejection fraction, but reduced systolic, diastolic, mean arterial, and left ventricular end diastolic pressures. These changes help preserve cardiac function. The mechanism of gliptin-mediated decrease in BP may be attributed in part to reduced plasma renin concentration and cardiac angiotensin II (Ang II) contents as well as increasing ACE2 and Angiotensin 1–7 (see Fig. 2). It can be also due to less atherosclerotic lesions in large arteries due to controlled lipotoxicity and CV lipid accumulation which is evident in many studies. Taken together, the studies presented in this review clearly found that gliptins improved systolic and diastolic left ventricular dysfunction in many CV disease models and so improved overall cardiac performance.

## Conclusions and prospective

Uncontrolled diabetes is associated with CV complications and predispose the patient to end-organ damage such as stroke and heart failure. Since their introduction into drug market, DPP4i have gained attention due to their effective glucose regulation and due to their beneficial CV effects. Studies on DPP4i proved that these effects are partly due to control of hyperglycemia and are also due to direct effect on the CV system via receptor-dependent and possibly receptor-independent effects too. DPP4i modulate not only the level of GLP-1, but also the concentration of other peptides that might exert vasoactive, and CV protective effects such as BNP, SDF-1α, and substance P. In this review, we summarized the result of most recent preclinical studies on CV protective effects of gliptins during the past 5 years. DPP4i control hyperglycemia, decreasing oxidative stress and inflammation, leading to less mitochondrial stress and cell death. They also enhance tissue repair and preserve endothelial function leading to improved myocardial perfusion. Moreover, DPP4i can significantly decrease cardiac Ang II but increase Ang1-7 which can also improve cardiac perfusion. These consistent findings in various CV diseases suggest promising cardioprotective potential of DPP4i, especially when considering their ability to improve glucose control without affecting body weight or causing hypoglycemia. However, further investigations on their mechanism and long-term safety data are required before recommending gliptins as CV-protecting agents in diabetes.
